# Adverse events of the yellow fever vaccine in chronic urticaria: evaluation of patients treated or not with omalizumab compared to healthy individuals^☆^^☆☆^

**DOI:** 10.1016/j.abd.2020.09.009

**Published:** 2021-05-15

**Authors:** Laura Ramos de Almeida, Roberta Fachini Criado, Paulo Ricardo Criado, Luis Felipe Ensina, Beatrice Martinez Zugaib Abdalla, Juarez Antônio Simões Quaresma

**Affiliations:** a Faculty of Medicine, Centro Universitário Saúde ABC, Santo André, SP, Brazil; b Division of Allergy, Clinical Immunology and Rheumatology, Department of Pediatrics, Universidade Federal de São Paulo, São Paulo, SP, Brazil; c Center for Biological and Health Sciences, Universidade Estadual do Pará, Belém, PA, Brazil

Dear Editor,

Currently, the treatment of Chronic Urticaria (CU) follows an international consensus. When CU does not respond to antihistamines for up to 4 weeks, the use of omalizumab is recommended for at least 6 months, at a dose of 300 mg subcutaneously every four weeks.^1^, ^2^

There is a formal contraindication for vaccination with an attenuated live virus in patients using immunobiological agents.

As of July 2017, in Brazil, there was an epidemic of Yellow Fever (YF) disease and the World Health Organization (WHO) recommended immunization with the Yellow Fever Vaccine (YFV) using the fractionated dose, on a temporary basis as a strategy to prevent the disease.^3^, ^4^, ^5^

As omalizumab acts exclusively on mast cell and basophil IgE and does not affect cell immunity, the study group recommended vaccination for YF in patients from areas at risk for the disease.^6^

The present study aimed to evaluate the occurrence of adverse reactions to YFV in patients with CU with and without the use of omalizumab and/or antihistamines. As a secondary objective, the reason for the non-vaccination of some patients using omalizumab was evaluated. A cross-sectional and observational study was conducted, by collecting demographic data from 89 patients who were being followed at the Urticaria Outpatient Clinic between January and April 2019.

A questionnaire was applied to patients with CU and healthy individuals, about adverse events to immunization, 30 days after the vaccination, and another questionnaire to patients with CU who did not receive the immunization, aiming to find out why they did not receive the YFV. Patients sign the free and informed consent form and the study was approved by the institution's research ethics committee under number 2,853,158.

The qualitative variables were shown as absolute and relative frequencies, whereas the quantitative variables were shown as mean, standard deviation, maximum and minimum values, using the normality test of Shapiro-Wilk (p > 0.05). Fischer's exact test was used to compare qualitative variables. For all analyses, the level of significance was set at α = 0.05. The statistical program Stata version 1 was used.

A total of 89 questionnaires were administered to patients with CU and 30 to healthy individuals who received immunization (Fig. 1). Table 1 shows the results of the demographic data, CU subtypes and the treatment of patients included in the study between January and April of 2019.

**Figure 1:**
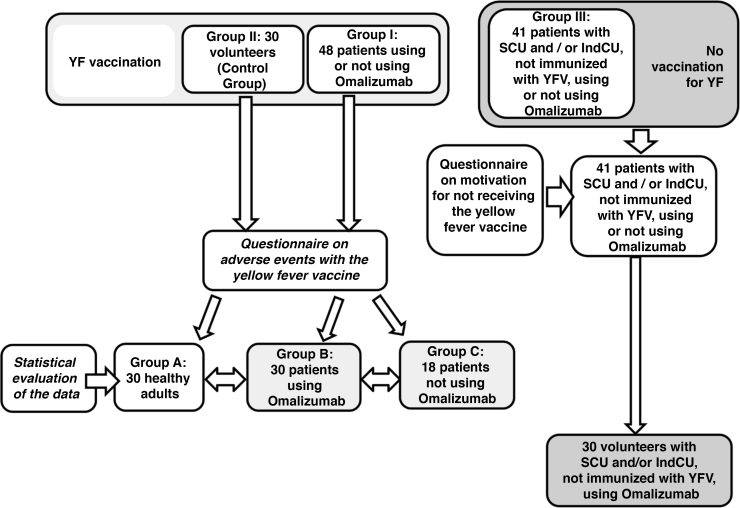
Data collection flowchart between CU patients immunized and not immunized with the Yellow Fever Vaccine (YFV) and healthy individuals immunized with the same vaccine.

**Table 1:** Demographic data, type of urticaria, use of omalizumab and immunization against YF among the 89 patients participating in the study.

Characteristics of patients	Frequency	%
Gender		
Male	15	16.85
Female	74	83.15
Urticaria classification		
SCU	71	79.78
IndCU	4	4.49
SCU plus IndCU	14	15.73
Clinical manifestations		
CU plus angioedema	63	70.79
Isolated CU	25	28.09
Isolated angioedema	1	1.12
Use of omalizumab		
No	35	39.33
Yes	54	0.67
Did you receive the yellow fever vaccine?		
No	41	46.07
Yes	48	53.93

SCU, spontaneous chronic urticaria; IndCU, inducible chronic urticaria.

Of the patients with CU, 48 (53.93%) received the YFV concomitantly with the treatment, whereas 41 (46.07%) did not receive it. All 48 patients who received the YFV answered the questionnaire about adverse reactions. Most patients (89.58%) did not report any adverse reactions to the YFV, concomitantly with the treatment with omalizumab. Three (6.25%) patients reported local reaction to the YFV, one (2.08%) reported asthenia and one (2.08%) reported exacerbation of a preëxistent angioedema (Table 2).

**Table 2:** Comparison between the report of adverse reactions attributed to vaccination against yellow fever, in healthy individuals (group A), patients with CU treated with omalizumab (group B), patients with CU without treatment with omalizumab (group C).

Groups of individuals	Adverse reactions attributed to yellow fever vaccine					Total
	None	Local cutaneous reaction	Asthenia	Angioedema exacerbation	Fever	
Grupo A	25 (83.33%)	3 (10.0%)	0 (0.0%)	0 (0.0%)	2 (6.67%)	30 (100%)
Grupo B	27 (90%)	1 (3.33%)	1 (3.33%)	1 (3.33%)	0 (0.0%)	30 (100%)
Grupo C	16 (88.89%)	2 (11.11%)	0 (0.0%)	0 (0.0%)	0 (0.0%)	18 (100%)
Total	68 (87.18%)	6 (7.69%)	1 (1.28%)	1 (1.28%)	2 (2.56%)	78 (100%)

In the group of 41 patients with CU who did not take the YFV, the majority (31.71%) did not seek immunization, as they had already been immunized prior to the CU treatment. A total of 26.83% reported “It was informed by another health professional that they should not take the vaccine because of the treatment”; 21.95% reported “They decided not to take it”; 12.20% stated, “I am afraid of taking the vaccine due to the disease”. The other patients answered the questionnaire with the following statements: “There was no time, I was advised not to get the vaccine because of bariatric surgery” and “I was advised not to take the vaccine due to pregnancy”, with each response comprising 2.44% of the answers.

Table 2 describes adverse events to YFV in 30 healthy individuals, 30 patients with CU treated with omalizumab and 18 patients with CU who were not treated with omalizumab.

The statistical analysis, using Fischer's exact test, showed no differences between the presence or absence of adverse reactions in groups A (healthy individuals), B (patients with CU treated with omalizumab) and C (patients with CU who were not treated with omalizumab), with a p-value of 0.553.

There are no reports in the literature regarding the use of omalizumab concomitantly with immunization against YF. Immunization against YF in patients with CU treated with omalizumab was advised because IgE-mediated reactions, mast cells and basophils, are not crucial to fight viral infections.

Only three patients (6.25%) mentioned a skin reaction at the vaccine injection site. The frequency of this event is 4% in the population receiving YFV.^6^ One individual (2.08%) reported asthenia. The occurrence of systemic manifestations is observed in 1% to 6% of individuals. Finally, one patient (2.08%) reported exacerbation of angioedema after taking the vaccine, which may be associated both with a possible reaction to YFV, and with the fluctuation in the urticaria control activity during treatment.

There was no statistical difference when comparing the groups of patients with CU using or not omalizumab who received the YFV. There was no statistical difference regarding the adverse effects of YFV in the control group of 30 healthy individuals when compared to the 30 patients with CU who were treated with omalizumab.

In conclusion, the present study confirmed that patients with CU undergoing treatment with antihistamines and/or omalizumab did not report adverse events after immunization against YF, when compared to healthy individuals, who received immunization in the same period, with the same vaccine provided by the Brazilian Ministry of Health.

## Financial support

None declared.

## Aurhors’ contributions

Laura Ramos de Almeida: Statistical analysis; drafting and editing of the manuscript; collection, analysis, and interpretation of data.

Roberta Fachini Criado: Approval of the final version of the manuscript; design and planning of the study; effective participation in research orientation.

Paulo Ricardo Criado: Approval of the final version of the manuscript; design and planning of the study; effective participation in research orientation.

Luis Felipe Ensina: Statistical analysis; approval of the final version of the manuscript; design and planning of the study; critical review of the literature.

Beatrice M. Z. Abdalla: Approval of the final version of the manuscript; drafting and editing of the manuscript; collection, analysis, and interpretation of data; critical review of the literature.

Juarez Antônio Simões Quaresma: Statistical analysis; approval of the final version of the manuscript; design and planning of the study; critical review of the literature.

## Conflicts of interest

None declared.
